# Venous arterial spin labeling MRI signal is associated with oxygen supply-independent reduction in cerebral oxygen extraction in typically aging older adults

**DOI:** 10.1162/IMAG.a.952

**Published:** 2025-10-17

**Authors:** Jan A. Kufer, Gabriele M. Gassner, Natalie S. Wheeler, Shrikanth M. Yadav, Riya Mittal, John Jacoby, Sarah F. Mellen, Katherine N. Maina, Nathaniel D. Mercaldo, David H. Salat, Meher R. Juttukonda

**Affiliations:** Athinoula A. Martinos Center for Biomedical Imaging, Department of Radiology, Massachusetts General Hospital, Charlestown, MA, United States; Department of Radiology, Harvard Medical School, Boston, MA, United States; Department of Neurology, University Hospital Zurich, Zurich, Switzerland; Neuroimaging for Veterans Center, Boston VA Healthcare System, Boston, MA, United States

**Keywords:** venous hyperintense signal (VHS), oxygen extraction fraction (OEF), arterial spin labeling (ASL), capillary dysfunction, aging

## Abstract

Microvascular flow disturbances may reduce capillary oxygen extraction efficiency and contribute to the accumulation of structural and functional pathology over the course of the aging process. Venous hyperintense signal (VHS) on arterial spin labeling (ASL) magnetic resonance imaging (MRI) has recently been proposed as a noninvasive marker of disturbed capillary flow patterns and may be associated with an oxygen supply-independent reduction in cerebral oxygen extraction fraction (OEF) in clinical populations. However, the role of VHS in typically aging older individuals remains under-investigated. Much physiologic inter-subject variability exists in OEF, primarily due to differences in cerebral hemodynamics (e.g., macro- and microvascular flow, transit times or blood velocity), and it is not well understood if these hemodynamic factors confound associations between VHS and OEF. Moreover, it remains unclear to what degree the appearance of VHS might be associated with imaging-related artifacts, including motion. To better characterize these effects, we conducted a multi-modal MRI study in a cohort of 36 typically aging older adults (60–80 years). We measured VHS, whole brain OEF, cerebral blood flow, microvascular oxygen supply and arterial transit times, and bulk flow in the internal carotid arteries and the superior sagittal sinus. Associations between cerebral hemodynamic physiology, head motion, VHS, and OEF were analyzed using combined multivariable and stepwise regression models. We found that VHS was associated with lower OEF and that VHS remained independently associated with reduced OEF in multivariable models adjusting for other vascular parameters. Importantly, VHS was not related to head motion. These findings suggest that VHS in older individuals may be indicative of distinct physiological mechanisms leading to reduced OEF, but further work is needed to directly elucidate their underpinnings.

## 1 Introduction

The human brain relies on oxidative metabolism for energy production, and this demand is supported by a steady supply of oxygen and nutrients delivered by the vascular system. The perfusion of brain tissue is characterized by cerebral blood flow (CBF), while the offloading of oxygen from the microvasculature into tissue is characterized by the oxygen extraction fraction (OEF). Impaired oxygen availability to brain tissue (as reflected by altered CBF and/or OEF) is thought to play an important role in aging and in the pathophysiology of cognitive decline and neurodegeneration, including Alzheimer’s disease (AD) (Damestani et al., 2024; Yang et al., 2023). In AD, increasing evidence suggests that hemodynamic alterations may, in some cases, prospectively drive (rather than simply represent a downstream consequence of) neurodegeneration (Wolters et al., 2017). In cognitively normal individuals, prior work has found elevated OEF (Lu & Ge, 2008) and higher cerebral metabolic rate of oxygen (CMRO_2_) with aging (Peng et al., 2014), indicating that OEF changes may reflect physiology beyond compensation for well-known age-related perfusion reductions (Juttukonda, Li, et al., 2021). Of note, age-related OEF increases were found to be more pronounced in those with higher vascular risk within a cohort of cognitively normal and mildly impaired subjects (Lin et al., 2023), while OEF may be distinctly reduced in some older individuals with low vascular risk and intact blood oxygen supply (Gassner et al., 2025). Neurodegeneration, including AD pathology, could result in OEF reductions secondary to lower energy consumption (Jiang, Lin, Liu, Sur, Xu, Hazel, Pottanat, Darrow, et al., 2020) although some studies also reported unchanged OEF with concomitant CBF reductions (Yang et al., 2023). In summary, these findings suggest that imaging markers related to cerebral oxygen metabolism could provide important insights into brain health.

Changes in OEF may be more completely understood by considering the contributions of capillary flow patterns (Hertz & Paulson, 1980; Kuschinsky & Paulson, 1992). Jespersen and Ostergaard (2012) have proposed that heterogeneous microvascular flow patterns may limit OEF and, thereby, tissue oxygenation and metabolism even in the absence of flow-limiting conditions. The effect is a result of the non-linearity of the well-known flow-diffusion equation, whereby CBF increases (i.e., transit time decreases) through any single capillary become disproportionately inefficient as a means of increasing tissue oxygen supply at higher flows (Buxton & Frank, 1997; Østergaard, 2020). It has been proposed that this effect may play a role in the pathogenesis and progression of age-related conditions, including AD (Eskildsen et al., 2017; Madsen et al., 2022; Nielsen et al., 2020; Østergaard et al., 2013) and small vessel disease (Dalby et al., 2019).

Recently, Juttukonda et al. (2019) proposed that the presence of venous hyperintense signals (VHS) on arterial spin labeling (ASL) magnetic resonance imaging (MRI) may serve as a qualitative and non-invasive indicator of microvascular flow disturbances that affect the efficiency of oxygen extraction. In ASL, perfusion-weighted images are generated by acquiring images of the brain with and without previous magnetic inversion of blood water spins while they pass through the cervical internal carotid arteries (ICA) and the vertebral arteries. After waiting a suitably long time (i.e., the post labeling delay [PLD]) to allow for these spins to arrive in the brain tissue, images are acquired with and without this ‘label’, and are subtracted and scaled to absolute units of CBF (Alsop et al., 2015). Under normal flow conditions, the majority of unextracted, magnetically labeled spins would be expected to arrive in the superior sagittal sinus (SSS) after a transit time of ~4500–4800 ms (Lin et al., 2018). Thus, the presence of signal hyperintensities (i.e., presence of labeled spins) inside of venous sinuses observed on perfusion-weighted ASL images acquired at a much earlier time (i.e., << 4500 ms) may indicate a shift of the global capillary transit time distribution curve toward lower transit times (i.e., change in mean transit time), broadening of the distribution with higher proportions of very low/high transit times (i.e., increased transit time heterogeneity), and/or changes in the water extraction fraction (Lin et al., 2018).

VHS effects have been previously investigated in patients with sickle cell disease (SCD) (Afzali-Hashemi et al., 2022; Juttukonda, Donahue, et al., 2021; Juttukonda et al., 2019; Stotesbury et al., 2022) as well as in aging (Juttukonda et al., 2022). In SCD, VHS was found to be associated with blood velocities in the cervical arteries (Juttukonda et al., 2019) and baseline CBF (Afzali-Hashemi et al., 2022). Lower OEF in the presence of VHS has been reported in sub-groups of adults with SCD and signs of structural damage (Juttukonda, Donahue, et al., 2021) as well as in older adults as a function of age (Juttukonda et al., 2022). In addition, Afzali-Hashemi et al. (2022) recently demonstrated that acetazolamide-induced increases in VHS might induce CMRO_2_ decreases, even after correcting VHS for CBF. Nevertheless, the overall physiological underpinnings of the VHS phenomenon and its relationship with other hemodynamic parameters remain incompletely understood, particularly in older subjects with presumably lower CBF, longer arterial transit times (ATT), and relatively normal blood hemoglobin concentrations. Moreover, contributions of imaging artifacts to VHS, for example, by head motion, have not been systematically evaluated and may be more pronounced in older individuals.

The overall purpose of this study was to investigate relationships between cerebrovascular physiology and VHS on ASL images in a cohort of typically aging adults in order to facilitate a deeper understanding of the physiologic changes as well as potential confounders that VHS in older age may originate from. Specifically, we hypothesized that VHS was related to cross-sectional OEF reductions beyond those expected by varying blood oxygen supply, and that the association was not related to common MRI artifacts, including head motion.

## 2 Methods

### 2.1 Study design, participants, and compliance with ethical standards

This study was approved by the Massachusetts General Hospital (MGH) Institutional Review Board and was performed in accordance with ethical standards for human subject research set forth under the Declaration of Helsinki. All subjects provided written, informed consent prior to participation. We recruited 45 typically aging adult volunteers (age range: 60–80 years) through the Brain Aging and Dementia lab research registry at MGH. Inclusion criteria consisted of (1) age-appropriate performance on a phone-administered neurocognitive screening exam and (2) the absence of major cardiovascular, neurological, or psychiatric disease. Participants underwent MRI, a thorough assessment of medical history and medication intake, and a physical and neuropsychological examination. Blood samples were obtained via venipuncture and used to measure individual hematocrit and hemoglobin concentration (Hb) values for physiological modeling. Of note, a few participants (n = 12) underwent more than one imaging session, in which case the first complete set of data acquired within a single session was used. Subjects without a complete set of hemodynamic parameters (see below) from at least one session were excluded (n = 9).

### 2.2 Magnetic resonance imaging

MRI data were acquired at 3 Tesla (Prisma; Siemens Healthcare; Erlangen, Germany) using a body transmit coil, 32-channel head receive coil, and software release VE11C. Unless stated otherwise, image processing was conducted using MATLAB R2023b (MathWorks, Natick, MA, USA) and FSL (FMRIB, Oxford, United Kingdom).

#### 2.2.1 Structural imaging

A 3D multi-echo magnetization-prepared rapid gradient echo (ME-MPRAGE) sequence using volumetric navigators (Tisdall et al., 2012; van der Kouwe et al., 2008) was acquired for co-registration and tissue segmentation: repetition time (TR) = 2500 ms, inversion time (TI) = 1000 ms, four evenly spaced echoes (TE_1_ = ΔTE = 1.8 ms), and flip angle (FA) = 8°, resolution = 0.8 mm isotropic. T_1_-weighted images were obtained by taking the root mean square across echoes and processed using the *recon-all* functionality in FreeSurfer (Dale et al., 1999; Fischl et al., 1999). In addition, T_1_-weighted images were processed to obtain individual segmentations of the dural venous sinuses (Puonti et al., 2020) using a development version of SAMSEG (Puonti et al., 2016) with the unprocessed images. SAMSEG is distributed within the FreeSurfer package, but runs separately from the *recon-all* pipeline. Of note, vein segmentation is supported by public SAMSEG distributions starting with FreeSurfer release 8.0.0, which was not yet available at the time of data preparation.

A 2D fluid-attenuated inversion recovery (FLAIR) sequence was acquired for segmentation of white matter hyperintensities: TR = 9000 ms, TI = 2500 ms, TE = 9.1 ms, FA 150°, and resolution 0.85 x 0.85 x 5 mm. FLAIR images were processed with the Lesion Segmentation Toolbox in SPM 12, using the ‘lesion prediction algorithm’ with subjects’ T_1_-weighted images for spatial corregistration. This yielded quantitative white matter lesion volumes (WMLV), which were subsequently log-transformed.

#### 2.2.2 Oxygenation imaging

T_2_-relaxation-under-spin-tagging (TRUST) (Lu & Ge, 2008) was used to quantify whole-brain OEF in the superior sagittal sinus. Briefly, the sequence employed a non-selective T_2_ preparation pulse to minimize the influence of flow effects on the T_2_ and a label-control image subtraction scheme analogous to ASL was applied to isolate signal originating from blood. Readout occurred at a plane placed approximately 20 mm superior to the confluence of sinuses. Images were acquired at four effective TEs (eTE = 0, 40, 80, and 160 ms), with three label-control pairs per eTE for averaging. An exponential decay curve was fit to these data points, and T_2,blood_ was obtained by adjusting the raw decay constant for hematocrit-corrected T_1,blood_. A calibration model derived specifically for human blood was then used to relate T_2,blood_ to the venous oxygen saturation Y_v_ (Bush et al., 2017). Finally, OEF was calculated as $\frac{Y_{a}-Y_{v}}{Y_{a}}$, with measured arterial oxygen saturation Y_a_.

#### 2.2.3 Perfusion imaging

Multi-delay pseudo-continuous arterial spin labelling (pCASL) was acquired for quantification of cerebral blood flow (CBF) and arterial transit times (ATT). The sequence utilized in this study was developed for the Lifespan Human Connectome Project in Aging (HCP). This implementation, which is referred to hereby as “HCP-ASL”, has been described in detail previously (Harms et al., 2018; Juttukonda, Li, et al., 2021); an up-to-date discussion of specific advantages and drawbacks of this sequence has recently been published (Kirk et al., 2025). Briefly, we acquired 43 pairs of label-control images (60 slices, resolution = 2.5 × 2.5 × 2.5 mm) without background suppression, with a labeling duration (LD) = 1500 ms, and five post-labeling delays (PLD_1_ = 200 ms, PLD_2_ = 700 ms, PLD_3_ = 1200 ms, PLD_4_ = 1700 ms and PLD_5_ = 2200 ms) and 2D multiband readout. An equilibrium magnetization (M_0_, TR = 15000 ms) image was acquired for CBF quantification in absolute units, and two spin-echo images with opposite phase encoding polarity were acquired to facilitate distortion correction. Multi-delay pCASL data were processed as described previously (Juttukonda, Li, et al., 2021). In brief, FSL *topup* was used for distortion correction using the spin echo images with opposite phase encoding polarity. Pairs of control-label images were subtracted and averaged for each PLD separately, and arterial transit times (ATT) calculated using a previously introduced two-stage cross-correlation approach (Juttukonda, Li, et al., 2021) according to the implementation described by Damestani et al. (Damestani et al., 2023). CBF values were then obtained using a two-compartment model with individual ATTs and T_1,blood_ (based on hematocrit), and by non-linear least-squares fitting of data from all PLDs. CBF and ATT values were extracted from cerebral gray matter using tissue masks obtained from FreeSurfer. Combining Y_a_, blood hemoglobin concentrations [Hb] and CBF averages in gray matter, we calculated DO_2_, assuming a hemoglobin oxygen-carrying capacity φ of 1.34 mL O_2_ per gram Hb according to the following equation (Gassner et al., 2025):

$$DO_{2}=\;\varphi \times Y_{A}\times [Hb]\times \;CBF$$

#### 2.2.4 Venous hyperintensity imaging

A separate single-PLD pCASL sequence with conventional 2D EPI readout was acquired for evaluation of venous hyperintense signal (VHS). The purpose behind a separate acquisition was to avoid potential contamination of VHS by large PLD variations in adjacent slices owing to the multiband readout of the HCP-ASL sequence. The sequence comprised 8 label-control pairs, LD = 1500 ms, PLD = 1200 ms, and an M_0_ image. Additional sequence parameters were as follows: TR = 4000 ms, TE = 12 ms, FA = 90°, resolution = 3.5 x 3.5 x 5 mm, 2D EPI readout, and 27 slices. The shorter-than-typically-recommended PLD (Alsop et al., 2015) was chosen to further sensitize the sequence for early venous arrival of labeled blood water and is consistent with our prior studies (Juttukonda et al., 2022). Label-control images were motion corrected and registered to the M_0_ image using FSL (Jenkinson et al., 2012) (‘mcflirt’; FMRIB Software Library), then pair-wisely subtracted, and averaged using FSL BASIL (Chappell et al., 2009) (‘asl_file’). Subsequently, kinetic fitting was performed with a two-compartment model and assuming age-appropriate values for ATT and the T_1_ of arterial blood, as well as standard values for labeling efficiency, T_1_ of brain tissue, and the blood-brain partition coefficient, as described before (Juttukonda et al., 2022). Finally, CBF images were registered to subjects’ individual T_1_-weighted images using FSL (‘flirt’). Average venous signal intensities were extracted from within a 12-voxels-wide, mid-sagittal portion of the abovementioned SAMSEG-segmented venous sinus mask, comprising the superior and inferior sagittal sinus, straight sinus, and confluence of sinuses. Note that VHS is shown nominally in units of mL/100g/min as the venous signal was extracted from the absolute CBF maps; however, these values should not be interpreted as perfusion since the quantification model is clearly not valid in macrovascular, venous voxels. For comparison, images were also independently rated twice by two raters (JK and MRJ) who were blinded to demographic information and using an ordinal three-point scale (no venous hyperintensity, focal venous hyperintensity, diffuse venous hyperintensity), as previously described (Juttukonda, Donahue, et al., 2021). Subsequently, the mode across the four ratings was taken; remaining disagreement was resolved by consensus. Moreover, we extracted an average gray matter CBF from these images and calculated a DO_2, Single-PLD_ as described above to test whether VHS and its possible effect on OEF was equally well explained by oxygen supply derived from this sequence. Additional measures were extracted from the quantified single-PLD ASL sequence to analyze potential confounding by motion. First, ‘fsl_motion_outliers’ was applied to each subject’s raw label-control series to obtain the mean framewise displacement (Power et al., 2012) between these images. Second, the quantified ASL signal from adjacent (i.e., within the same 12 voxels-wide mid-sagittal slab) gray matter, cerebrospinal fluid, and skull bone regions—also produced by SAMSEG—was extracted to evaluate the spatial specificity of VHS. The specificity would be reduced by partial volume effects, which are increased by motion.

#### 2.2.5 Phase contrast MRI

2D phase contrast MRI was obtained of (a) the neck arteries above the carotid bifurcation at approximately the location of the ASL labeling and (b) the superior sagittal sinus, approximately 20 mm superior to the confluence of sinuses at the location of the TRUST acquisitions with the following sequence parameters: TR = 21 ms, TE = 7 ms, FA = 10°, field of view (FOV) = 220 × 220 mm, resolution = 0.5 × 0.5 mm, and velocity encoding (VENC) = 40 cm/s. Blood vessels of interest were manually segmented by drawing a polygonal ROI (using *polyroi* in MATLAB) onto the PC images to delineate the left and right internal carotid arteries, and the sagittal sinus, respectively. Despite efforts to omit the vertebral curvatures (similarly as for the ASL planning), the vertebral arteries were not well discernable or not imaged perpendicularly in n = 7 cases and therefore omitted from the main analysis to avoid further reduction of the sample size. Supplemental sensitivity analyses in the subset with complete cervical phase contrast measures (n = 29) did not yield meaningfully different results with respect to VHS effects (Supplementary Fig. S3; Supplementary Table S1). Cross-sectional areas of vessels were calculated from these ROIs, and phase information was converted to blood velocities using the known VENC. Means of blood velocities were calculated across voxels comprising each ROI. Blood flow through these vessels was then calculated as the product of the cross-sectional area with the blood velocity average, each. Total flow and cross-sectional area of the ICAs were calculated as the sum of left and right ICA values, and a mean velocity in the ICAs calculated by dividing total flow by the total cross-sectional area. Our rationale for including the various phase-contrast MRI parameters was as follows: For SSS cross-sectional areas and blood velocities, we hypothesized that these local SSS conditions could directly and artifactually influence both TRUST and single-PLD ASL signals, for example, by contributions of SSS cross-sectional areas to partial volume effects in VHS, thereby acting as confounders. Furthermore, SSS velocities might be distinctly sensitive to venous transit times, which could reveal a contribution to VHS not expected to relate to oxygen extraction differences and that should be accounted for, if applicable. In addition, given that OEF was a global measure of oxygenation in the superior sagittal sinus, we rationalized that flow derived at the same location might more closely reflect perfusion in the brain tissue with venous drainage to the SSS, that is, the same location that OEF is measured at. Hence, SSS flow could explain additional physiological OEF variability that might otherwise be misattributed to VHS or, alternatively, obscure associations between OEF and VHS. For ICA phase-contrast measurements, we rationalized that derived flows could contain perfusion information that is obscured in the microvascular ASL measures due to unaccounted variability in labeling efficiencies in the cervical arteries of different subjects. ICA velocities are also expected to reflect large vessel arterial transit times which are different from ASL-based ATTs that partially reflect microvascular transit times. As such, a significant portion of inter-subject VHS variability unrelated to oxygen extraction could be explained by ICA velocities, and teasing these influences apart from capillary transit time effects would be expected to yield additional insights. Lastly, ICA cross-sectional areas were included as they might be independently associated with blood pressure (Krejza et al., 2006), which has been shown to relate to OEF as well (Jiang, Lin, Liu, Sur, Xu, Hazel, Pottanat, Yasar, et al., 2020).

### 2.3 Statistics

Statistical analyses were performed using MATLAB R2023b. Normal distribution of the data was analyzed using the Shapiro-Wilk test. For regression models, normality and homoscedasticity of residuals was analyzed visually by QQ-Plots and by plotting residuals against fitted values. Statistical significance was assumed at p < 0.05 and, where appropriate, Bonferroni correction was employed to account for multiple comparisons. For comparisons between subgroups with and without radiological evidence of VHS, we evaluated differences in continuous demographic variables using Welch’s t-test (normally distributed variables) or non-parametric Mann-Whitney U tests (non-normally distributed variables). Fisher’s exact test and a chi^2^ test were used to investigate distributions of sex and race in the sample respectively.

First, we fitted a linear regression model for the association between VHS and OEF. Second, we investigated associations between VHS, OEF, and head motion as quantified by framewise displacement. To this end, a bivariate regression model involving framewise displacement and VHS, as well as a multivariable model of the combined effect of VHS and framewise displacement on OEF were computed. Additionally, linear regressions between OEF and adjacent GM, CSF and bone ASL signal averages from the single-PLD sequence, each, were obtained. Subsequently, pair-wise multivariable models for the combined effects of OEF-related parameters were computed. This served to establish whether confounding by partial volume effects influenced some of the univariate associations. Third, we investigated interrelations between OEF, VHS, and other hemodynamic-vascular parameters described above. *Pearson* correlations were pair-wisely obtained and the associated p-values, with and without correcting for multiple comparisons using Bonferroni corrections, were summarized in a correlation matrix. Moreover, linear multivariable regression models involving pair-wise combinations of hemodynamic parameters with OEF as the outcome were computed. Lastly, stepwise regression employing MATLAB’s *stepwiselm* function using default settings for adding/removing variables and limited to linear terms (i.e., no interactions, quadratic terms, etc.) was run with OEF as the dependent variable and all mentioned variables (hemodynamics, framewise displacement, local GM/CSF/bone signals) as candidate predictors. Thereby, we aimed to establish whether VHS was independently associated with reduced OEF, even after accounting for combinations of multiple other potentially explanatory variables. We reasoned that this would determine if VHS had a direct association with OEF or is merely an epiphenomenon arising from high flow, blood oxygen supply, pre-/postcapillary blood velocities, and/or lower arterial transit times.

## 3 Results

### 3.1 Participant demographics

Demographic data for individuals with and without VHS on single-delay ASL maps (based on qualitative evaluation by raters) are listed in Table 1. Those with radiological evidence of VHS had higher quantitative venous sinus ASL signal (p < 0.001) and lower OEF (p = 0.02) than those without; however, only the difference in VHS survived Bonferroni correction (p_corr_ < 0.05). Additionally, we observed non-significant tendencies for higher DO_2, Single-PLD_ (p = 0.05) and SSS velocities/flows (p = 0.06/0.05) in these subjects.

**Table 1.** Participant Demographics by visual presence/absence of VHS.

		Total (n = 36)	VHS absent (n = 13)	VHS present (n = 23)	p-value
Age	(years)	68.1 ± 5.4	67.9 ± 5.9	68.2 ± 5.3	0.88
Sex	(% female)	58.3	69.2	52.2	0.48
Race	(% Asian)	11.1	15.4	8.7	0.11
	(% Black)	13.9	30.8	4.3	
	(% White)	72.2	53.8	82.6	
	(% Other)	2.8	0	4.3	
APOE	(% ε4 carrier)	33.3	46.2	26.1	0.28
Hb	(g/dL)	13.9 ± 1.3	13.8 ± 0.9	14.0 ± 1.5	0.72
Hct	(%)	41.7 ± 3.2	41.8 ± 2.1	41.7 ± 3.7	0.96
WMLV	(log mL)	0.10 ± 1.63	0.10 ± 1.71	0.10 ± 1.62	0.99
Brain vol.	(cm^3^)	1047 ± 109	1018 ± 69	1064 ± 125	0.17
GM vol.	(cm^3^)	581 ± 56	564 ± 30	591 ± 65	0.11
ST vol.	(cm^3^)	923 ± 98	898 ± 61	937 ± 112	0.19
VHS	(mL/100g/min)	97.6 ± 28.8	72.1 ± 14.4	112.0 ± 24.6	<0.001*
OEF	(%)	37.1 ± 5.4	40.4 ± 6.7	35.2 ± 3.5	0.019
CBF	mL/100g/min	68.1 ± 11.0	66.1 ± 9.6	69.2 ± 11.8	0.39
ATT	(s)	1.35 ± 0.21	1.39 ± 0.23	1.32 ± 0.20	0.49
DO_2_	(mLO_2_/100g/min)	12.4 ± 1.8	12.0 ± 1.9	12.6 ± 1.8	0.36
DO_2, Single-PLD_	(mLO_2_/100g/min)	8.7 ± 1.5	8.0 ± 1.6	9.1 ± 1.3	0.05
SSS Area	(cm^2^)	0.25 ± 0.05	0.25 ± 0.05	0.25 ± 0.06	0.91
SSS Velocity	(cm/s)	18.2 ± 5.0	16.2 ± 4.7	19.4 ± 4.9	0.06
SSS Flow	(mL/min)	269 ± 63	240 ± 67	285 ± 56	0.05
ICA Area	(cm^2^)	0.35 ± 0.07	0.34 ± 0.07	0.36 ± 0.06	0.31
ICA Velocity	(cm/s)	20.0 ± 3.9	19.7 ± 3.4	20.2 ± 4.2	0.67
ICA Flow	(mL/min)	418 ± 82	394 ± 80	432 ± 82	0.19

Continuous variables are presented as mean ± standard deviation. APOE = apolipoprotein E. Hb = blood hemoglobin concentration. Hct = blood hematocrit. WMLV = white matter lesion volume (log-transformed). Brain vol. = brain volume, excluding ventricles. GM vol. = total gray matter volume. ST vol. = supratentorial volume, excluding ventricles. OEF = oxygen extraction fraction. DO_2_ = oxygen supply from sequences noted in subscript. ATT = arterial transit time. SSS = superior sagittal sinus. ICA = internal carotid arteries. Differences remaining statistically significant after Bonferroni correction (p_corr_ < 0.05) are marked by an asterisk.

### 3.2 Venous hyperintense signal data and univariate association with OEF

**Fig. 1.**
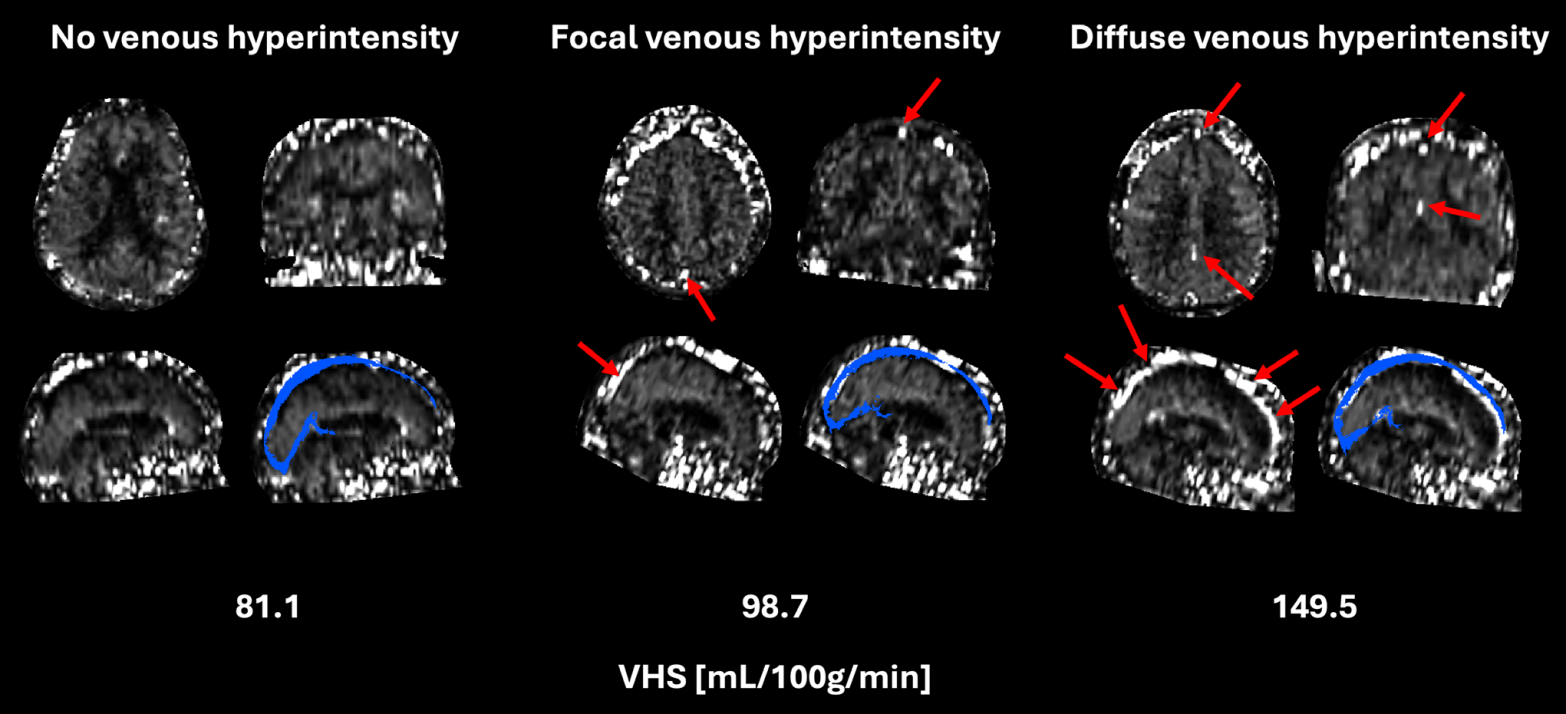
Exemplary slices showing different ASL images with different degrees of venous hyperintense signal (VHS). From left to right, images from individuals with no venous hyperintensity, focal VHS, and diffuse VHS are displayed. Numbers below images indicate quantitative venous ASL signal in nominal units of mL/100g/min. Blue overlays on sagittal slices show individual venous sinus mask for the extraction of venous ASL signal. Red arrows highlight visual appearance of VHS. Note that a mask including the entire head (skull, scalp, eyes, etc.) rather than a brain mask was applied; this served to enable assessment and confirmation of a clear distinction between visual VHS and adjacent noise signals.

**Fig. 2.**
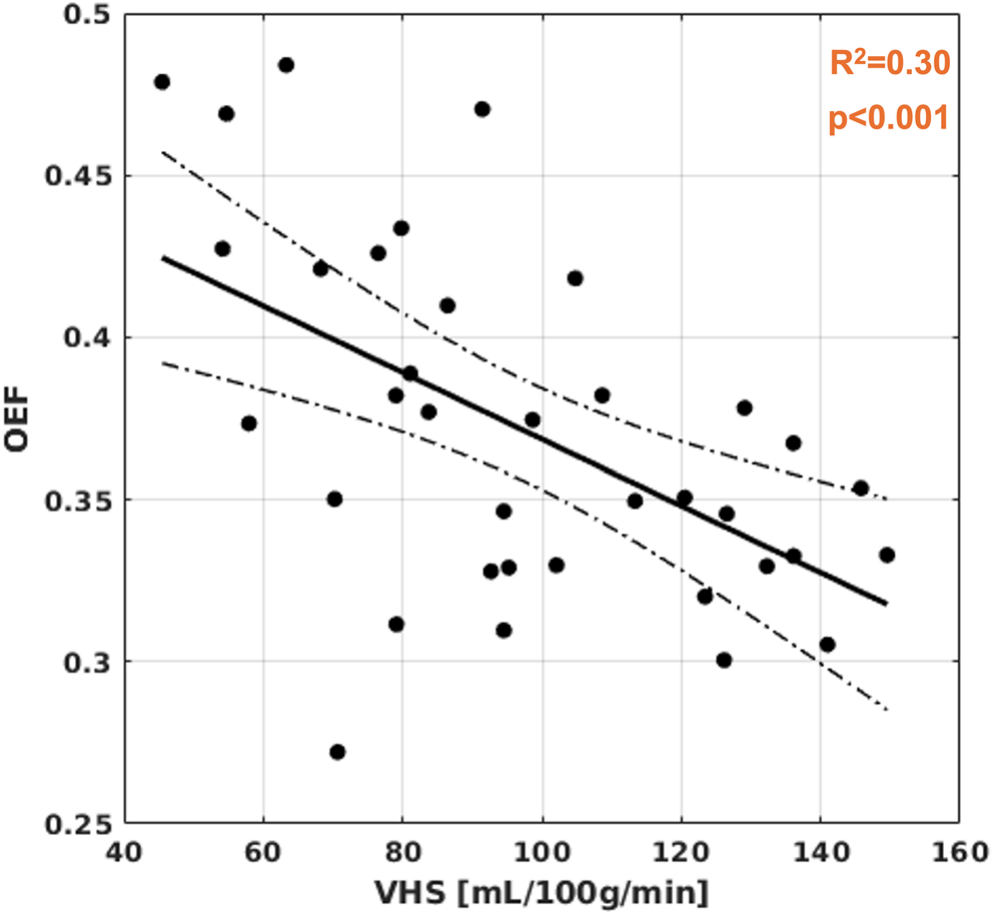
Association of VHS with OEF. Black dots correspond to per-subject parameter averages. A significant negative correlation was observed between VHS and OEF, consistent with the hypothesis that the shunting of labeled blood water spins (i.e., early arrival on ASL images) might be reflective of inefficient capillary oxygen extraction.

Exemplary single PLD ASL images with different degrees of visual evidence for VHS, and of the individual venous sinus masks are shown in Figure 1. Prior to analyzing cerebral microvascular physiology, we confirmed good agreement between quantitative venous ASL signal and visual appearance of VHS (Supplementary Fig. S1; Kendall’s tau = 0.52, p < 0.001), as observed similarly before (Juttukonda et al., 2019). Interestingly, some individuals with comparably long mean arterial transit times and minor arterial transit artifacts also exhibited VHS; exemplary images for these cases are shown in Supplementary Figure S2. Figure 2 shows the bivariate linear association between VHS and OEF; a significant negative relationship was observed (R^2^ = 0.30, p < 0.001).

### 3.3 Effects of head motion on VHS and its association with OEF

**Fig. 3.**
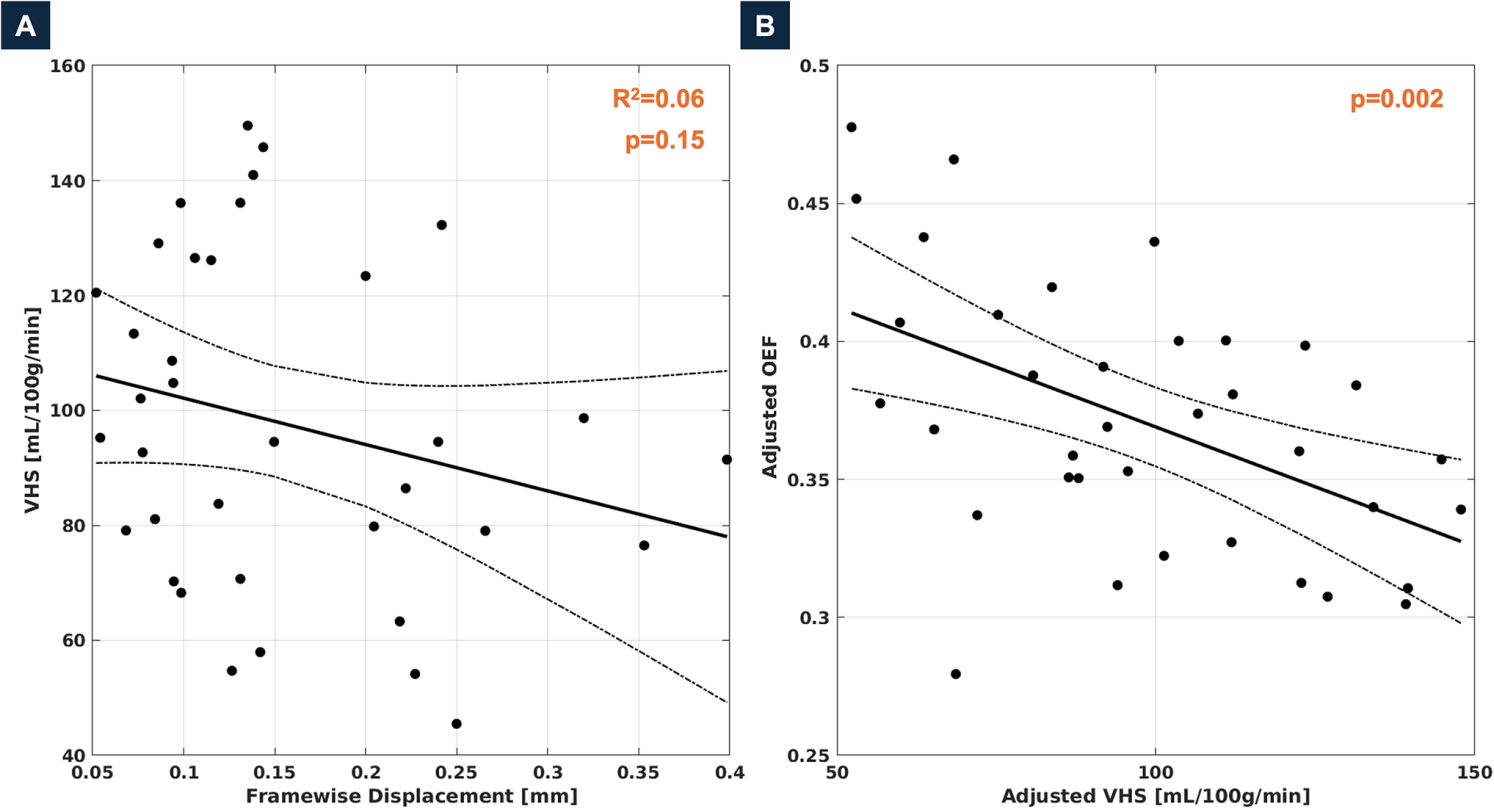
Effect of head motion on VHS (A) and the association between VHS and OEF (B). The scatter plot in (A) shows the association between per-subject head motion as quantified by framewise displacement in the single-PLD ASL label-control series (i.e., the sequence that VHS was quantified from) and VHS across participants. No significant relationship was observed (p = 0.15). The added variable plot in (B) illustrates the association between VHS and OEF adjusting for framewise displacement as a potential confounder. Higher VHS remained significantly associated with reduced OEF (p < 0.01).

**Fig. 4.**
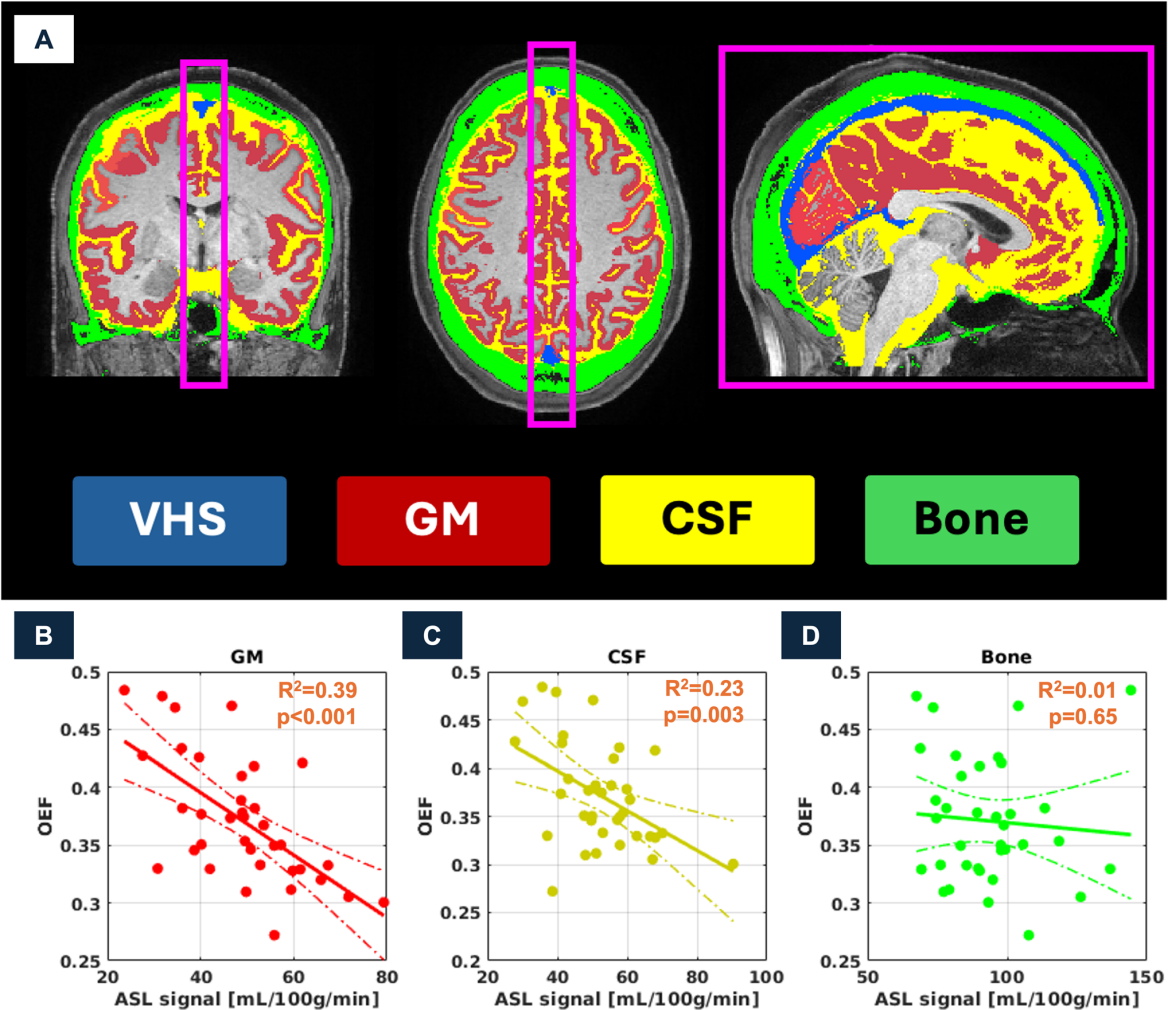
Evaluation of associations between OEF and single-PLD ASL signal in VHS-adjacent regions. Top row (A) shows exemplary individual SAMSEG-derived gray matter (GM, (B)), cerebrospinal fluid (CSF, (C)), skull bone (D) as well as, for reference, VHS (blue) segmentations overlaid onto the T_1_-weighted image of an exemplary subject. Mean ASL signal was extracted from each of these regions within the mid-sagittal slab (illustrated by the pink box) and correlated with OEF across subjects (bottom row). Note that, as for VHS, the ASL signal for CSF and bone is units of pseudo-CBF, that is, they were extracted from a quantitative CBF map in [mL/100g/min], although the underlying model is not meaningful in these regions. Significant negative correlations, similar to VHS, were observed for GM and CSF but not bone signals.

Figure 3 illustrates the effect of motion in the single-PLD ASL sequence, as quantified by framewise displacement, on VHS. No significant correlation between VHS and framewise displacement was observed (R^2^ = 0.06, p = 0.15). Correspondingly, the negative association between VHS and OEF remained significant when controlling for motion (p = 0.002). Additionally, we examined more indirect effects of motion through their contribution to partial volume effects by comparing the association observed between VHS and OEF to associations between OEF and single-PLD ASL signals extracted from neighboring regions of interest, that is, cortical GM, CSF, and skull bone, all from the same 12 voxels-wide mid-sagittal slab that the venous sinus mask was restricted to (Fig. 4, region of analyses illustrated as pink box). Similar to VHS, we observed significant negative associations with OEF for GM signal (R^2^ = 0.40, p < 0.001) and CSF signal (R^2^ = 0.23, p = 0.003). However, subsequent multivariable analysis (Table 2) suggests that the association between OEF and CSF was confounded by GM signal (i.e., partial voluming), with CSF signal becoming non-significant (p = 0.81). Similarly, CSF signal was not a significant predictor of OEF after controlling for VHS (p = 0.26). However, GM signal (p = 0.002) and VHS (p = 0.029) remained simultaneously associated with OEF, corresponding to independent effects of VHS on OEF not explained by confounding through partial voluming with adjacent GM signal (i.e., CBF), including through head motion.

**Table 2.** Pairwise multivariable regression models for the effect of OEF-related single-PLD ASL measures in Figure 4 on OEF.

	Estimate	Standard Error	T statistic	p-value
GM	-0.0021	0.0006	-3.33777	0.002
VHS	-0.0006	0.0003	-2.2776	0.029
CSF	-0.0009	0.0008	-1.1361	0.26
VHS	-0.0008	0.0004	-2.1393	0.040
GM	-0.0029	0.0010	-2.9794	0.005
CSF	0.0002	0.0010	0.2483	0.81

Associations between CSF and OEF seen in the univariate regression in Figure 4 vanish after controlling for the adjacent GM or VHS signal, indicating partial voluming. ASL signals in venous sinuses (i.e., VHS) and adjacent GM contained complementary information.

### 3.4 Associations among hemodynamic parameters and pair-wise joint effects on OEF

**Fig. 5.**
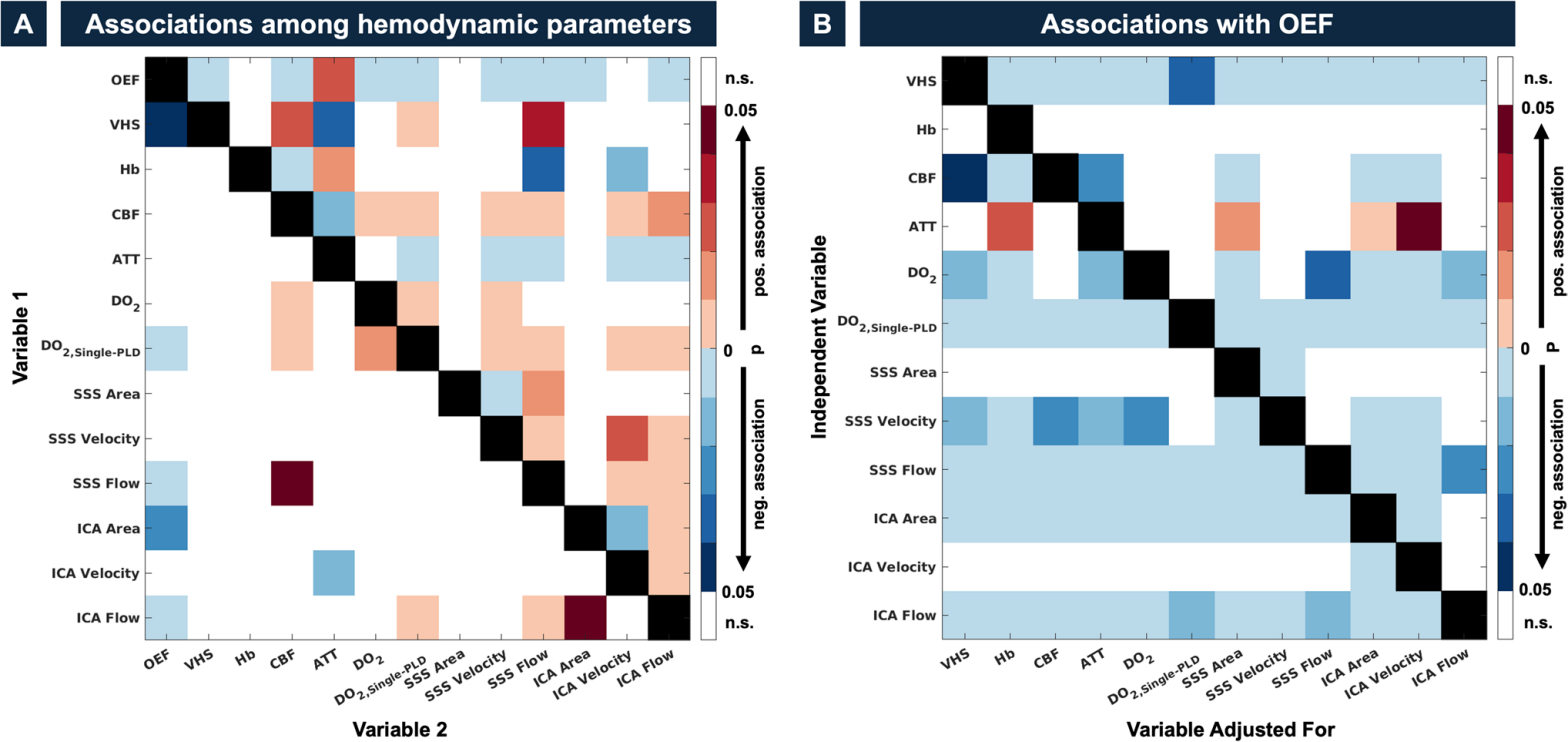
Interrelations among parameters of cerebrovascular physiology (A) and pair-wisely adjusted associations with OEF (B). The correlation matrix in (A) shows p-values for *Pearson* correlations between hemodynamic parameters in the study. Positive and negative associations are shown in red and blue colors, respectively. Tiles above the autocorrelation line (black tiles, toward the top-right corner) show uncorrected p-values, while those below the autocorrelation line (toward the bottom-left corner) show Bonferroni-corrected values (n = 78 statistical tests). Note that the univariate correlation between VHS and OEF survived Bonferroni correction. The correlation matrix in (B) visualizes effects of hemodynamics on OEF (i.e., dependent variable) under pairwise adjustment for other parameters of cerebrovascular physiology. The y-axis indicates the independent variable for which the adjusted p-value is shown, while the x-axis indicates the confounder being controlled for. Note that VHS remains significantly negatively associated with OEF adjusting for any of the other variables (B, top line).

First, we investigated associations among OEF, VHS, and other hemodynamic parameters (Fig. 5A). The link between VHS and OEF (see Fig. 2) survived Bonferroni-correction for the large number (n = 78) of independent correlations among hemodynamic variables (p_corr_ = 0.04). Before adjusting for multiple comparisons, OEF was correlated with all measures of perfusion/oxygen supply (CBF, DO_2_, DO_2, Single-PLD_, SSS, and ICA flows) as well as with ATTs, but only DO_2, Single-PLD_ and ICA/SSS flows survived Bonferroni correction. Besides OEF, VHS was negatively associated with ATT (p = 0.02) and Hb (p = 0.08), and positively correlated with CBF, DO_2, Single-PLD_, and SSS flow (p < 0.05, each). However, no associations between these hemodynamic parameters and VHS remained significant after adjusting for multiple comparisons. After Bonferroni correction, correlations within ASL-based perfusion/oxygen supply measures (CBF, DO_2_, DO_2, Single-PLD_) and within phase-contrast flow measures remained mutually significant (p_corr_<0.05, each). Furthermore, CBF was significantly associated with ICA flow, and DO_2, Single-PLD_ with SSS flows. Lastly, we observed a noteworthy strong correlation between ATTs and ICA velocities (p_corr_ = 0.017).

Second, we aimed to elucidate if some the associations between hemodynamic parameters and OEF were confounded by related vascular parameters (Fig. 5B). Importantly, we found that none of the hemodynamic parameters explained the negative association between VHS and OEF, as shown by sustained significance under pair-wise adjustment (all p < 0.05). The weak positive association between ATT and OEF became non-significant after adjusting for any of the perfusion-related parameters. Interestingly, DO_2, Single-PLD_ and ICA/SSS phase contrast flows, as well as ICA areas, all seemed to reflect mutually exclusive information about OEF not explained by any other vascular measure (except for joint ICA area/flow).

### 3.5 Stepwise regression model of OEF

The last analysis in this study employed stepwise regression to explore if more complex combinations of methodological (framewise displacement; adjacent GM, CSF, or skull single-PLD ASL signals) and/or physiological (CBF, ATT, DO_2_, DO_2, Single-PLD_, ICA/SSS flow, velocities, and cross-sectional areas) sources of noise or confounding could explain the observed inverse association between VHS and OEF. The resulting model is summarized in Table 3. Higher VHS magnitudes were independently associated with reduced OEF (β = -0.0004, p = 0.017) adjusting for all other relevant predictors of OEF. DO_2, Single-PLD_ (p < 0.001), SSS flow (p = 0.019), and ICA cross-sectional areas (p < 0.001) were retained as additional predictors.

**Table 3.** Stepwise regression model with OEF as the outcome.

	Estimate	Standard Error	T statistic	p-value
Intercept	0.7218	0.0316	22.87	<0.001
VHS	-0.0004	0.0002	-2.53	0.017
DO_2, Single-PLD_	-0.0161	0.0035	-4.63	<0.001
SSS Flow	-0.0002	0.0001	-2.47	0.019
ICA Area	-0.3310	0.0669	-4.95	<0.001

## 4 Discussion

In this study, we comprehensively characterized the physiological relationships between venous hyperintense signal on arterial spin labeling MRI (i.e., VHS), oxygen extraction (i.e., OEF), and other microvascular (cerebral blood flow/oxygen delivery and arterial transit times) and macrovascular (flow, blood velocities, and cross-sectional areas of the internal carotid arteries and the superior sagittal sinus) hemodynamic physiology. We also investigated effects of MRI artifacts, including motion and partial volume effects, on the OEF-VHS relationship. The overall goal was to disentangle the effect of VHS on OEF from potential confounding effects of these other factors. As hypothesized, we found that individuals with increased VHS exhibited reduced OEF. Crucially, although VHS and OEF were both associated with hemodynamic-vascular parameters, including oxygen supply, CBF, or transit times/blood velocities, pairwise multivariable regression revealed that the effect of VHS on OEF was not confounded by any of these parameters. Moreover, head motion did not confound VHS. Critically, in a combined stepwise regression model, VHS remained independently associated with reduced OEF, suggesting that VHS may be independently connected to lower OEF. Thus, VHS in typically aging older adults may reflect physiological alterations that contribute to lower OEF than expected for the given arterial supply of oxygen.

### 4.1 Associations between VHS, OEF, and cerebrovascular physiology

Our study in a cohort of older, typically aging individuals revealed an inverse association between VHS and OEF. This finding is consistent with—although in itself does not proof—the previously reported hypothesis that VHS might indicate impaired capillary oxygen extraction (Afzali-Hashemi et al., 2022; Juttukonda, Donahue, et al., 2021; Juttukonda et al., 2019, 2022; Song et al., 2025), through mechanisms yet to be elucidated. This negative association between VHS and OEF agrees with several previous studies. In individuals with sickle cell disease, Juttukonda et al. found an unfavorable effect of VHS on OEF, and this effect was more pronounced in patients with structural damage (i.e., white matter hyperintensities and silent cortical infarcts) than in those without (Juttukonda, Donahue, et al., 2021). However, in the same study, a small effect for lower OEF in those with VHS as well as a relatively high prevalence of VHS (62%) was also observed in the healthy control group, suggesting that this physiological phenomenon may not be limited to those with overt disease. Similarly, another study in SCD observed an inverse correlation between VHS and CMRO_2_, across both healthy control participants and adult SCD patients (Afzali-Hashemi et al., 2022). In typically aging healthy individuals of older age, a recent study likewise suggested a link between VHS and reduced oxygen extraction (Juttukonda et al., 2022), where oxygen extraction efficiency as estimated from contrast perfusion MRI (Jespersen & Østergaard, 2012) was inversely correlated with older age in older participants with visual evidence of VHS. The opposite was found in participants without VHS. This latter study highlighted the potential utility of VHS in conditions other than SCD. However, the present study is the first to quantify venous ASL signals in typically aging older adults to demonstrate a direct inverse association between this signal and OEF.

In line with previous studies (Juttukonda, Donahue, et al., 2021), we also observed associations between VHS and perfusion-related parameters (Fig. 5A). Specifically, we found positive associations between VHS and CBF, DO_2, Single-PLD_ and SSS flow, as well as a negative correlation between VHS and ATT. Of note, previous studies in SCD patients also found links between ICA blood velocities and VHS (Juttukonda et al., 2019), which we did not observe and that might be specific to the pathomechanism present in SCD. In any case, these associations highlighted that VHS does not purely reflect reduced OEF, but is strongly weighted by perfusion. According to the central volume theorem (and absent major compensatory increases in blood volume), transit times and blood flow are expected to change in opposite directions. Therefore, in individuals with higher flow, the overall transit time distribution (covering vascular transit from the labeling plane to the venous sinuses) is expected to be shifted toward shorter transit times. Under the hypothesis that the measured venous ASL difference signal, indeed, originates from labeled spins, this would explain parallel increases in perfusion and VHS. Importantly, this perfusion dependency of VHS may complicate the interpretation of cross-sectional associations between VHS and OEF. Indeed, we observed similar inverse associations between OEF and DO_2_, CBF and macrovascular flows, in agreement with known cerebrovascular physiology. According to Fick’s law, the product between DO_2_ (which, in turn, depends on CBF/flow, Hb and Y_a_) and OEF determines the steady-state consumption of oxygen that can be supported. Across brain regions, CBF is tightly coupled to regional CMRO_2_ and, consequently, OEF is typically relatively spatially uniform (Hyder et al., 2015). Across individuals, on the other hand, OEF has been found to be inversely related to CBF or DO_2_, presumably to maintain a narrower range of CMRO_2_ (Germuska et al., 2019), which our cross-sectional study confirmed. OEF, thus, appeared to be changing in opposition to DO_2_, to at least partly mitigate inter-subject oxygen supply variability not due to different oxygen demand. Jiang, Lin, Liu, Sur, Xu, Hazel, Pottanat, Yasar, et al. (2020) have shown that a substantial portion of inter-subject variability in OEF (~50%) may be due to differences in end-tidal CO_2_, which is well known to elicit CBF and, thereby, DO_2_ increases. In summary, results of our univariate analyses confirmed that VHS was negatively associated with OEF, but also that—due to confounding of this relationship by perfusion—such univariate analyses do not directly indicate to what degree VHS-accompanying OEF reductions reflect physiologic adaptation to perfusion states/oxygen supply (reflecting the perfusion component of VHS) versus perfusion-independent mechanisms reducing OEF (and, consequently, CMRO_2_), including putative oxygen extraction inefficiency (i.e., a capillary dysfunction or flow disturbance component of VHS). As proposed by Afzali-Hashemi et al. (2022), we attempted to isolate potential perfusion-unrelated contributions to VHS by combined regression models, that evaluated the association of VHS with OEF adjusting for hemodynamic-vascular confounders, as discussed below in Section 4.3.

### 4.2 Effects of imaging artifacts on VHS and its association with OEF

Besides perfusion and oxygen supply, MRI artifacts may also contribute to VHS and potentially confound its association with OEF. First, we evaluated whether imaging artifacts, in particular head motion, could explain VHS and confound the association between VHS and OEF. Head motion during acquisition of the ASL label-control series is known to result in hyperintense subtraction artifacts surrounding the brain in derived CBF maps (Clement et al., 2022). We did not observe a correlation between framewise displacement in the single-PLD ASL raw images and VHS (Fig. 3A); consequently, VHS remained significantly associated with OEF after adjusting for framewise displacement (Fig. 3B). This finding agrees well with the significant association between quantitative venous signal (i.e., VHS) and qualitative evaluation of hyperintensity by visual ratings (Supplementary Fig. S1). Raters explicitly distinguished hyperintensities likely due to motion artifacts from contiguous, sharply demarcated hyperintensities in the venous sinuses deemed to represent VHS. Moreover, we observed that VHS exhibited a unique association with OEF that was not similarly observed for ASL signal extracted from segmented regions of interest in the immediate vicinity of the major veins (Fig. 4). In these regions, namely cortical bone and CSF, no or relatively little (for CSF) (Jiang, Lin, Liu, Sur, Xu, Hazel, Pottanat, Yasar, et al., 2020; Petitclerc et al., 2023) physiologic signal is expected. Indeed, in cortical bone we observed no relationship between signal on the ASL image and OEF, whereas for CSF we found a similar negative association with OEF as for VHS. However, in contrast to VHS, this association did not survive adjustment for GM signal (and adjustment for VHS), indicating partial volume effects. Furthermore, the effect of VHS on OEF was independent of the spatially adjacent GM signal, indicating that it was not purely confounded by partial voluming with tissue CBF. Increased partial volume effects would also be expected in the case of motion within individual label/control images leading to blurring of tissue boundaries; such effects cannot be removed using the typical motion correction approaches in image processing. In summary, we did not find evidence that VHS itself or its association with OEF was caused or confounded by imaging artifacts related to head motion.

### 4.3 Combined model of OEF

Finally, we investigated the independence of VHS effects on OEF from other physiological confounders by means of combined pair-wise regressions as well as a stepwise regression model. Following a published approach (Afzali-Hashemi et al., 2022), we rationalized that a residual negative relationship between VHS and OEF after adjustment for confounders could lend preliminary support to the hypothesis that VHS partly reflects reduced oxygen extraction efficiency. In pair-wise regression models, adjustment for any of the potential confounders did not render the association between VHS and OEF non-significant (Fig. 5B). Similarly, Azali-Hashemi et al. previously reported that an unfavorable effect of VHS on oxygen metabolism prevailed after adjusting for perfusion (Afzali-Hashemi et al., 2022), in agreement with our finding that VHS was not merely an epiphenomenon of high CBF/DO_2_. However, our study observed this effect for baseline VHS/OEF and in older individuals, rather than in healthy and SCD-affected younger adults in response to acetazolamide-induced CBF increases (Afzali-Hashemi et al., 2022). Moreover, stepwise regression retained VHS as a variable significantly related to OEF (Table 3), together with DO_2, Single-PLD_, SSS flow, and ICA cross-sectional areas as independent variables. Taken together, these findings provided reasonable evidence that VHS could be associated with reduced OEF independently even of combinations of more than one of the other factors under consideration. Furthermore, candidate variables for stepwise regression also included the MRI artifacts from Figures 3 and 4, but none of them were selected for inclusion into the stepwise regression model. We, thus, carefully interpret our findings as an indication that VHS, even in older individuals, might be independently associated with reduced OEF. This independent association might reflect a mechanism that requires further elucidation but could reflect reduced extraction efficiency due to microvascular flow disturbances such as elevated capillary transit time heterogeneity, which is known to affect tissue oxygenation independently of perfusion (Jespersen & Østergaard, 2012). Alternatively, microvascular shunting in older adults could arise from a reduction in capillary density, that is, reduced microvascular blood volume, which would result in reduced mean transit times for the same CBF. Moreover, the dynamics of venous arterial spin labeling signal might be related to blood brain barrier integrity through the water extraction fraction (Lin et al., 2018); however, in this case, the biophysical relationship to reduced oxygen extraction appears less clear. While future studies will need to elucidate the relative contributions of these factors, any of them or their combination could uncouple VHS from CBF/DO_2_ and thus explain the presence of VHS in older adults despite typically lower perfusion and prolonged arterial transit times in these cohorts (Juttukonda, Li, et al., 2021). Moreover, given that VHS is a global marker, prolonged arterial transit to some brain regions could be present in parallel with reduced overall transit times through other supply regions of the vascular tree. In addition or alternatively, microvascular transit time heterogeneity could result in the early arrival of some labeled blood even if overall flow is reduced, potentially to the degree where this effect overcomes the VHS reduction expected with reduced perfusion. Importantly, the presence of VHS in older individuals has been recently described in two independent samples using analogous sequence timings, including in a larger lifespan cohort that, notably, observed VHS-exhibiting individuals to be of older age than those without VHS (Juttukonda et al., 2022; Michael et al., 2025). Moreover, studies in younger cohorts with and without SCD independently observed cases of VHS comparable to SCD patients in some healthy control subjects, despite CBF differences of 50–100% between groups (Afzali-Hashemi et al., 2022; Juttukonda, Donahue, et al., 2021). Given that these differences significantly exceeded the expected age-related CBF decline and concomittant ATT increase (Juttukonda, Li, et al., 2021), they support the notion that VHS might be sufficiently independent of global perfusion to be observed in older adults. Nevertheless, additional studies using the same sequence and quantitative evaluation of VHS, including with adjustment for CBF/DO_2_, across a wide age range will be needed to support strong conclusions about the dynamics of VHS over the lifespan.

The additional, independent association of DO_2_ with reduced OEF was expected due to reasons discussed above; however, it is interesting that two correlated measures of oxygen supply (DO_2, Single-PLD_ and SSS flow) both survived the stepwise model selection procedure. Since oxygen supply calculated from venous phase-contrast MRI measurement should most closely reflect oxygen availability conditions in the tissue draining into the SSS, where OEF was measured by TRUST MRI, it is plausible that SSS flow emerged as a particularly informative predictor of OEF. Given that DO_2, Single-PLD_, which was obtained from the same scan as VHS, was included in the combined model, it is less likely that the effect of VHS on OEF is due to partial voluming with gray matter signal (already included in DO_2, Single-PLD_) or because it would simply reflect perfusion otherwise. Lastly, although unrelated to VHS, an interesting finding was the emergence of ICA cross-sectional areas as a strong predictor of OEF. We speculate that this relationship could be related to blood pressure, which has been reported to relate both to differences in cervical artery diameters (Krejza et al., 2006) and to inter-subject OEF variability (Jiang, Lin, Liu, Sur, Xu, Hazel, Pottanat, Yasar, et al., 2020). However, this latter finding was not reproduced in the supplemental sensitivity analysis including the vertebral arteries in the subgroup of subjects with complete data; as such, it should be interpreted with caution.

### 4.4 Limitations

First, the cross-sectional and observational design of this study limited our ability to draw causal inferences. In particular, it was difficult to directly establish whether any observed alterations in hemodynamic parameters indicated limitations of CMRO_2_ due to insufficiency of the particular cerebrovascular physiological state or if these parameters were secondarily regulated towards a lower demand for oxygen. We posit that the former is the case, since our subjects did not present with overt functional impairments or significant atrophy as would be the case in manifest neurodegenerative disease. Second, the sample size was modest, which may limit the generalizability of our findings. In addition, the limited sample size did not allow us to consider confounding or differential effects by age, sex, and/or race. However, the age range was narrow (60–80 years) by design, limiting the potential impact differences in age may incur. Third, the TRUST technique only yielded regional OEF values from the sagittal sinus and since venous drainage territories are variable and difficult to determine, we were unable to obtain CBF/DO_2_ estimates from precisely those brain regions draining into the SSS or to normalize ICA/SSS flows to this exact brain volume. We aimed to partly mitigate this by simultaneously considering the global flow measurements by phase-contrast MRI in stepwise regression; nevertheless, the inherent ROI-dependency of averaged ASL measures complicates the interpretation of their association with globally measured OEF. Fourth, as a whole-brain measure, VHS is a semi-quantitative marker weighted by both perfusion information and putative microvascular flow disturbances, and does not directly quantify capillary dysfunction in absolute units such as, for example, voxel-wise capillary transit time heterogeneity derived from contrast perfusion MRI (Mouridsen et al., 2014). Novel techniques for regional OEF quantification within the venous sinuses (Lin et al., 2024) may enable probing the VHS-OEF association in a more regionally resolved fashion in future studies. Fifth, we investigated the interrelations between VHS, OEF, and cerebrovascular physiology within a (multi-)linear framework. In particular, the true relationship between higher CBF and higher VHS could be non-linear, including through compounding effects of reduced microvascular water extraction, decreased T_1_ decay of spins arriving to the veins, and altered venous T_2_ relaxation through decreased OEF. In this case, linearly adjusting for perfusion/oxygen supply might not have been sufficient to examine independent effects of VHS on OEF. Future development of quantitative signal models for venous ASL signal for the employed sequence together with simulations might be helpful to investigate this further. Lastly, the current study utilized a single-PLD ASL sequence for VHS assessment, and future studies should employ suitable multi-PLD ASL sequences to obtain a deeper understanding of the relationship between VHS and transit times, especially in older subjects with prolonged ATTs and simultaneous VHS.

## 5 Conclusion

This study identified that mean arterial spin labeling signal in the major venous sinuses (i.e., VHS) may serve as a potentially independent marker of reduced oxygen extraction, unexplained by variations in macro- and microvascular hemodynamics and potentially reflecting capillary dysfunction.

## Supplementary Material

Supplementary Material

## Data Availability

Data and/or code that support the presented results will be made available to qualified investigators upon reasonable request made to the corresponding author. Data access will be through an access-controlled repository and facilitated through a formal data-sharing agreement.
