# Supplementary material for: Venous arterial spin labeling MRI signal is associated with oxygen supply-independent reduction in cerebral oxygen extraction in typically aging older adults
Source: Imaging Neurosci (Camb). 2025 Oct 17;3:IMAG.a.952. doi: 10.1162/IMAG.a.952 (PMC12534710; doi:10.1162/IMAG.a.952)
Supplement: Supplementary Material [file IMAG.a.952_supp.pdf]

Supplemental Material for "Venous arterial spin labeling MRI signal is associated with oxygen supply-independent reduction in cerebral oxygen extraction in typically aging older adults"

Jan A. Kufer[1,2], Gabriele M. Gassner[1,3], Natalie S. Wheeler[1], Shrikanth M. Yadav[1], Riya Mittal[1], John Jacoby[1], Sarah F. Mellen[1], Katherine N. Maina[1], Nathaniel D. Mercaldo,[2] David H. Salat[1,2,4], Meher R. Juttukonda[1,2]

[1]Athinoula A. Martinos Center for Biomedical Imaging, Department of Radiology, Massachusetts General Hospital, Charlestown, MA, USA

[2]Department of Radiology, Harvard Medical School, Boston, MA, USA

[3]Department of Neurology, University Hospital Zurich, Zurich, Switzerland

[4]Neuroimaging for Veterans Center, Boston VA Healthcare System, Boston, MA, USA

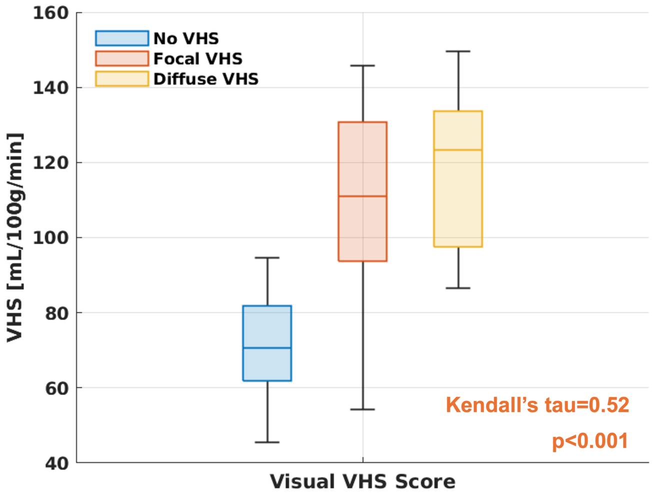

<u>Supplemental Figure 1</u>: Association of quantitative VHS with qualitative ratings on a categorical, ordinal scale. We observed a statistically significant monotonic trend, associating no, focal and diffuse visual presence of venous hyperintensities to gradually increasing quantitative VHS (Kendall's tau=0.52, p<0.001).

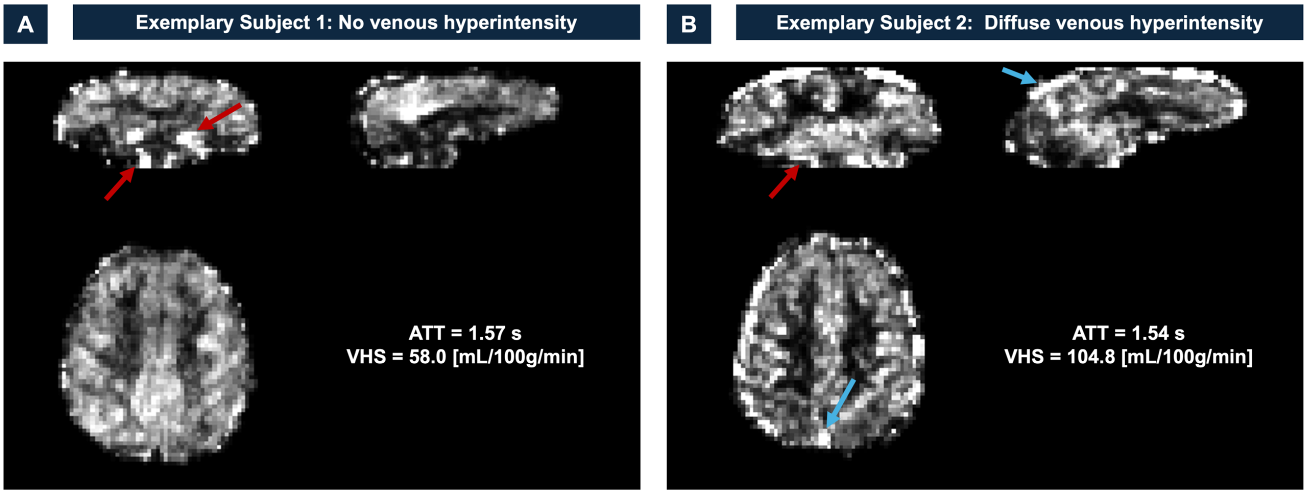

<u>Supplemental Figure 2</u>: Exemplary images from two subjects with (A) and without (B) VHS in the presence of comparably long ATT exceeding the post-labeling delay (PLD = 1200 ms) of the single-PLD ASL sequence. On both images, some arterial transit artifacts (ATA) are visible (red arrows). While the subject in (A) does not qualitatively show hyperintense signal in the venous sinuses, such venous hyperintense signal is present in subject 2 (B, blue arrows), with corresponding observations for quantitative VHS. Note that, different from Figure 1 in the main text, maps are shown in native space and after applying a brain mask, and have been smoothed and are scaled differently to highlight ATAs and VHS simultaneously.

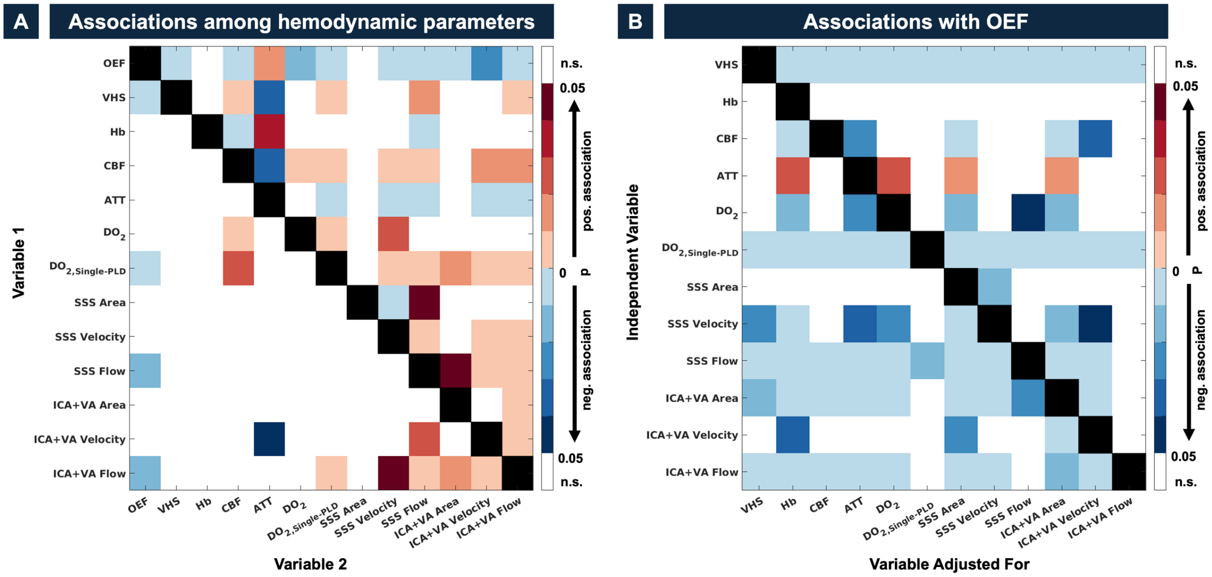

<u>Supplemental Figure 3</u>: Interrelations among parameters of cerebrovascular physiology (A) and pair-wisely adjusted associations with OEF (B) in the subset of individuals (n=29) with phase contrast MRI of both internal carotid arteries (ICA) and vertebral arteries (VA). The correlation matrix in (A) shows p-values for *Pearson* correlations between hemodynamic parameters in the study. Positive and negative associations are shown in red and blue colors, respectively. Tiles above the autocorrelation line (black tiles, towards the top-right corner) show uncorrected p-values, while those below the autocorrelation line (towards the bottom-left corner) show Bonferroni-corrected values (n=78 statistical tests). Note that the univariate correlation between VHS and OEF survived Bonferroni correction. The correlation matrix in (B) visualizes effects of hemodynamics on OEF (i.e., dependent variable) under pairwise adjustment for other parameters of cerebrovascular physiology. The y-axis indicates the independent variable for which the adjusted p-value is shown, while the x-axis indicates the confounder being controlled for. Note that VHS remains significantly negatively associated with OEF adjusting for any of the other variables (B, top line).

<u>Supplemental Table 1</u>: Stepwise regression model with OEF as the outcome in the subset of individuals (n=29) with phase contrast MRI of both internal carotid arteries (ICA) and vertebral arteries (VA). VHS = venous hyperintense signal. DO_2, Single-PLD = oxygen supply calculated from single-PLD ASL sequence (same as VHS). SSS = superior sagittal sinus. ICA = internal carotid arteries. VA = vertebral arteries. Hb = blood hemoglobin concentration.

	Estimate	Standard Error	T statistic	p value
Intercept	0.7708	0.0669	11.51	<0.001
VHS	-0.0006	0.0002	-3.44	0.002
$DO_{2,\ Single\text{-}PLD}$	-0.0184	0.0035	-5.28	<0.001
SSS Flow	-0.0004	0.0001	-4.00	<0.001
ICA + VA Velocity	0.0042	0.0021	2.04	0.053
Hb	-0.0108	0.0037	-2.95	0.007
